# Night shift work and inflammatory markers in male workers aged 20–39 in a display manufacturing company

**DOI:** 10.1186/s40557-016-0135-y

**Published:** 2016-09-20

**Authors:** Seong-Woo Kim, Eun-Chul Jang, Soon-Chan Kwon, Wook Han, Min-Sung Kang, Young-Hyeon Nam, Yong-Jin Lee

**Affiliations:** 1 Department of Occupational & Environmental Medicine, Soonchunhyang University Cheonan Hospital, 31, Suncheonhyang 6-gil, Dongnam-gu, Cheonan-si, Chungcheongnam-do 31151 Republic of Korea; 2 Environmental Health Center for Asbestos, Soonchunhyang University Cheonan Hospital, 67, Suncheonhyang 3-gil, Dongnam-gu, Cheonan-si, Chungcheongnam-do 31151 Republic of Korea

**Keywords:** Shift work, Inflammation, High sensitivity C-reactive protein, Leukocyte count, White blood cell

## Abstract

**Background:**

This study aimed to determine the association between shift work and inflammatory markers, which are independent risk factors of cardiovascular diseases, in male manual workers at a display manufacturing company.

**Methods:**

This study was conducted between June 1 and July 31, 2015 on 244 male manual workers aged 20–39 years old at a display manufacturing company and investigated age, marital status, education level, alcohol consumption habit, smoking habit, regular exercise habit, sleep duration, sleep debt, sleep insufficiency, past medical history, current and past shift work experience, duration of shift work, and weekly work hours through face-to-face interviews using structured questionnaires and performed blood tests. Study participants were divided into daytime, former shift, and current shift workers based on the work schedule. Chi-square tests and one-way analyses of variance were performed to compare inflammatory markers and cardiovascular disease risk factors, and analyses of covariance were conducted after adjusting for variables potentially affecting inflammatory markers.

**Results:**

High-sensitivity C-reactive protein (hs-CRP; mean ± standard deviation) levels in daytime, former shift, and current shift workers were 0.65 ± 0.43, 0.75 ± 0.43, and 0.86 ± 0.72 mg/L, respectively (*p* = 0.029). The leukocyte count (mean ± standard deviation) was 5,556 ± 1,123, 6,210 ± 1,366, and 6,530 ± 1,216 cells/μL, respectively (*p* < 0.001). Both hs-CRP level and leukocyte count were significantly higher in current shift workers than in daytime workers, and leukocyte count was higher in former shift workers than in daytime workers. After adjusting for variables potentially affecting inflammatory markers, hs-CRP levels (adjusted mean ± standard deviation) in daytime and current shift workers were 0.59 ± 0.06 and 0.92 ± 0.07 mg/L, respectively (*p* = 0.002). The leukocyte count (adjusted mean ± standard deviation) was 5,557 ± 124 and 6,498 ± 144 cells/μL, respectively (*p* < 0.001).

**Conclusions:**

A significant association between shift work and increases in inflammatory markers was confirmed. Because chronic low-grade inflammation plays an important role in the development of cardiovascular diseases, regular follow-up of inflammatory markers as a marker of cardiovascular diseases in shift workers may serve as an early indicator in predicting the effects of shift work on health.

## Background

In the few thousand years of the history of mankind, overnight activities were limited to particular groups, such as soldiers or sailors. The wide distribution of electricity enabled activities during night time much like during daytime; hence, the life of mankind started to undergo significant changes, one of which was a gradual increase in shift work due to social, technical, and economic needs [1, 2].

Shift work encompasses all types of work that take place outside regular daytime hours (between 7 am and 6 pm) [3]. The demand for shift work has been increasing globally, with 15–20 % of all workers in many industrial countries employed in shift work [4]. In the USA, according to the Bureau of Labor Statistics in 2004, 14.8 % of all workers were employed in shift work, including night work [5]. According to the 6th European Working Conditions Survey from 2015, 21 % of all workers in 35 European countries were employed in shift work, while 19 % were employed in night work [6]. In Korea, according to the results of a pilot survey of the 2012 Enterprise Labor Cost Survey from the Ministry of Employment and Labor, 15.5 % of all companies (89,582 samples) and 33.6 % of companies with 30 workers or more had a shift work system [7].

The working hours of shift workers, which do not match with their body and daily life rhythms, exert negative influences on the health of shift workers. Effects of shift work on health include cardiovascular diseases (CVDs), such as angina pectoris and myocardial infarction (MI) [8, 9]; cerebrovascular diseases, such as stroke [10]; metabolic syndrome [11]; mental illnesses, such as depression [12]; sleep disorders [13]; gastrointestinal disorders [14]; breast cancer [15]; and prostate cancers [16]. In relation to cancer, in 2007 the International Agency for Research on Cancer categorized shift work, including night work, as a Group 2A probable carcinogen to humans [5].

Research on the association between shift work and CVD has been carried out for several decades. Bøggild et al. conducted a meta-analysis of 17 epidemiological studies and reported a 40 % increase in the risk of CVD in shift workers compared to that in daytime workers [8]. Recently, Vyas et al. performed a meta-analysis of 34 studies and reported 23 and 24 % increases in the incidence of MI and coronary heart disease (CHD), respectively, in shift workers compared to that occurring in daytime workers [9]. A 15-year follow-up study on 504 paper mill workers in Sweden showed that the relative risk of CHD increased 1.5-fold, 2.0-fold, 2.2-fold, and 2.8-fold in individuals who had been employed in shift work for 2–5 years, 6–10 years, 11–15 years, and 16–20 years, respectively; thus, there was a dose-response relationship between the duration of shift work and the risk of CHD [17].

Atherosclerosis, an underlying pathology of CHD, was formerly considered a simple accumulation of lipids on vascular walls; however, it is now recognized as an inflammatory disease caused by a complex interaction between vascular endothelial cells, immune cells, and vascular smooth muscle cells; moreover, chronic low-grade inflammation induces CVD by playing an important role in the initiation and progression of atherosclerosis and destabilization and rupture of atherosclerotic plaques [18, 19]. Typical indicators of such inflammatory condition include high-sensitivity C-reactive protein (hs-CRP) and leukocyte count, both of which are strong predictors of CVD [19, 20].

hs-CRP and leukocyte count were significantly higher in shift workers than in daytime workers, suggesting that inflammation is a possible pathway linking shift work and CVD [21]. However, most studies were conducted outside of South Korea, and South Korean reports analyzing the association between shift work and inflammatory markers are largely lacking. Therefore, the current study was conducted to confirm the association between shift work and inflammatory markers in South Korea.

## Methods

### Study participants

This study was conducted at a university hospital from June 1 to July 31, 2015. From a display manufacturing company located in Cheonan, Chungcheongnam-do, 244 male manual workers aged 20–39 years old were selected as study participants after exclusion of 30 workers based on history of infectious diseases within the past 3 months; history of CVD, thyroid diseases, liver diseases, kidney diseases, autoimmune diseases, or cancer. This study was approved by the Institutional Review Board of Soonchunhyang University Hospital, Cheonan (IRB No. 2015-04-005-003), and all participants provided signed, informed consent after receiving an explanation regarding the study objectives and methods.

### Study methods

We performed face-to-face interviews using a structured questionnaire consisting of questions addressing general characteristics of the participants, such as age; marital status; education level, alcohol consumption; smoking; regular exercise; past medical history on CVD and chronic diseases; sleeping habit, such as sleep duration, sleep debt, and frequency of sleep insufficiency; and job-related details such as current and past shift work experience, duration of shift work, weekly work hours, and shift work characteristics (shift pattern, direction of shift rotation, and regularity).

Marital status was categorized as married or other (not married, divorced, or widowed). Education level was categorized as 12 years or less or more than 12 years. According to their alcohol consumption habits, subjects were categorized as “drinker” (those that consume alcohol at least once a week) or “non-drinker” (others that consume alcohol less frequently than once a week). Smoking habit was categorized as “smoker” or “non-smoker.” Regular exercise habit was categorized as “regular exercise” (those that exercise three times or more per week) or “non-regular exercise” (others that exercise less than three times per week). Sleep duration was defined as the average daily sleep duration for the past month, sleep debt as the difference in hours between obtained sleep and desired sleep needed to stay alert the next day for the past month, and the frequency of sleep insufficiency as the number of days per week on which the participants perceived, based on their subjective feeling, that they did not get enough rest or sleep within the past month.

The display manufacturing company at which the present study was conducted employed a four-crew/three-shift work system rotating counterclockwise regularly (the following sequence every 24 days: 6 night shifts, 2 days off, 6 evening shifts, 2 days off, 6 day shifts, 2 days off). Participants were categorized as daytime workers (currently working from 7–8 am to 5–6 pm with no previous shift work experience), former shift workers (currently working as a daytime worker but with previous shift work experience, including night work), or current shift workers (currently working as a shift worker, including night work).

Weight and height were measured using an automated height and weight measuring device (BSM330, Biospace, Korea) in an upright position and light apparel without shoes, and body mass index (BMI) was calculated by dividing the weight (kg) by the square of height (m^2^). Waist circumference was measured in an upright position at the midpoint between the lowest rib and the iliac crest after a quiet exhalation. Blood pressure was measured in a seated position using a mercury sphygmomanometer after at least 10 min of rest.

Blood was drawn between 7:30 am and 10:00 am after confirming that the participants fasted for at least 12 h. In particular, tests on shift workers were only conducted when they did not have an overnight shift on the previous day. Fasting blood sugar (FBS), triglycerides (TG), high density lipoprotein cholesterol (HDL-C), low density lipoprotein cholesterol (LDL-C), uric acid, hs-CRP, and glycated hemoglobin (HbA1c) were measured using an automated chemistry analyzer (COBAS 8000 & COBAS 800 INTEGRA, Roche Diagnostics, Tokyo, Japan), and leukocyte count was determined using an automatic blood cell analyzer (Sysmex XE-2100, Sysmex Corporation, Kobe, Japan).

### Statistical analysis

Distribution of participant characteristics is presented as frequency and percentage, and continuous variable data are shown as mean ± standard deviation. As hs-CRP and leukocyte count did not follow a normal distribution, they were analyzed after log transformation. Participants were divided into daytime workers, former shift workers, and current shift workers based on the work schedule, and chi-square tests and one-way analysis of variance were used to compare the general characteristics, occupational characteristics, and blood test results. To confirm the association between inflammatory markers (hs-CRP level and leukocyte count) and CVD risk factors, age-adjusted correlation analysis was conducted. Further, after adjusting for factors reported to affect inflammatory markers, such as age [22], BMI [23], waist circumference [23], alcohol consumption [24], smoking [25], regular exercise [23], sleep duration [26], sleep debt [27], sleep insufficiency [26], education [28], and weekly work hours [29], differences in blood inflammatory markers caused by different work patterns were investigated using analysis of covariance. Statistical analysis was performed using SPSS version 19.0 software (SPSS, Inc., Chicago, IL, USA), and statistical significance was set at a *p*-value of <0.05.

## Results

### General and occupational characteristics of study participants

The proportion of daytime workers (45.9 %) was the highest, followed by current shift workers (35.2 %) and former shift workers (18.9 %). The smoking rate was significantly higher in current shift workers than in daytime workers and in former shift workers. The proportion of daytime workers who received more than 12 years of education was significantly higher than that in former shift workers and current shift workers. No significant difference between the groups was observed for age, marital status, alcohol consumption habit, regular exercise habit, sleep duration, and weekly work hours. Sleep debt and sleep insufficiency were significantly higher in current shift workers than that in daytime workers (Table 1). The average duration of shift work for current shift workers and former shift workers was 9.95 ± 2.99 years and 3.46 ± 2.79 years, respectively (data not shown).

**Table 1:** General and occupational characteristics of study participants*

Variables	Work schedule group (*n* = 244)			*p*-value^†^
	Daytime (*n* = 112)	Former shift (*n* = 46)	Current shift (*n* = 86)	
Age (years)	32.2 ± 4.3	32.9 ± 2.9	33.3 ± 3.5	0.091
Marital status				
Married	62(55.4)	28(60.9)	57(66.3)	0.296
Other	50(44.6)	18(39.1)	29(33.7)	
Education level (years)				
≤ 12	8(7.1)	9(19.6)	22(25.6)	0.002
> 12	104(92.9)	37(80.4)	64(74.4)	
Alcohol consumption				
Non-Drinker	23(20.5)	6(13.0)	18(20.9)	0.493
Drinker	89(79.5)	40(87.0)	68(79.1)	
Smoking				
No	100(89.3)	37(80.4)	64(74.4)	0.023
Yes	12(10.7)	9(19.6)	22(25.6)	
Regular exercise				
No	9(8.0)	5(10.9)	11(12.8)	0.543
Yes	103(92.0)	41(89.1)	75(87.2)	
Sleep duration (hours/day)	6.33 ± 0.92	6.25 ± 1.04	6.47 ± 0.93	0.381
Sleep debt (hours/day)	1.21^a^ ± 0.93	1.39 ± 0.91	1.56^a^ ± 1.01	0.037
Sleep insufficiency (days/week)	1.47^a^ ± 1.43	1.81 ± 1.75	2.07^a^ ± 1.43	0.019
Weekly work hours				
< 48	25(22.3)	12(26.1)	13(15.1)	0.267
≥ 48	87(77.7)	34(73.9)	73(84.9)	

^*^values are mean ± standard deviation or number(%)

^†^compared using chi-square test or one-way analysis of variance

^a^same letters indicate statistical significance

### Anthropometric and biochemical characteristics of study participants

For current shift workers and daytime workers, the systolic blood pressure (SBP) was 128.1 ± 9.1 mmHg and 124.8 ± 9.7 mmHg, respectively (*p* = 0.046), the diastolic blood pressure (DBP) was 80.9 ± 6.7 mmHg and 77.0 ± 7.8 mmHg, respectively (*p* = 0.001), and hs-CRP was 0.86 ± 0.72 mg/L and 0.65 ± 0.43 mg/L, respectively (*p* = 0.029); all of SBP, DBP, and hs-CRP were significantly higher in current shift workers than in daytime workers. Similarly, for current shift workers, daytime workers, and former shift workers, the FBS was 96.0 ± 11.9 mg/dL, 90.1 ± 7.7 mg/dL, and 91.1 ± 8.4 mg/dL, respectively (*p* < 0.001), while HbA1c was 5.48 ± 0.33, 5.30 ± 0.24, and 5.32 ± 0.25 %, respectively (*p* < 0.001); current shift workers showed significant increases in FBS and HbA1c over daytime workers and former shift workers. Leukocyte count was 6,530 ± 1,216 cells/μL for current shift workers, 6,210 ± 1,366 cells/μL for former shift workers, and 5,556 ± 1,123 cells/μL for daytime workers (*p* < 0.001), with current shift workers and former shift workers showing significantly higher counts compared to daytime workers. BMI, waist circumference, TG, HDL-C, LDL-C, and uric acid did not show significant differences between the groups (Table 2).

**Table 2:** Anthropometric and biochemical characteristics of study participants^*^

Variables	Work schedule group (*n* = 244)			*p*-value^†^
	Daytime (*n* = 112)	Former shift (*n* = 46)	Current shift (*n* = 86)	
BMI (kg/m^2^)	24.2 ± 2.8	24.0 ± 2.4	24.4 ± 2.5	0.629
WC (cm)	82.9 ± 6.5	81.5 ± 7.1	83.6 ± 6.4	0.216
SBP (mmHg)	124.8^a^ ± 9.7	126.5 ± 7.6	128.1^a^ ± 9.1	0.046
DBP (mmHg)	77.0^a^ ± 7.8	77.8 ± 6.6	80.9^a^ ± 6.7	0.001
FBS (mg/dL)	90.1^a^ ± 7.7	91.1^b^ ± 8.4	96.0^a,b^ ± 11.9	<0.001
HbA1c (%)	5.30^a^ ± 0.24	5.32^b^ ± 0.25	5.48^a,b^ ± 0.33	<0.001
TG (mg/dL)	113.1 ± 74.2	133.9 ± 74.5	137.5 ± 84.0	0.068
HDL-C (mg/dL)	53.8 ± 12.1	50.0 ± 13.3	50.5 ± 11.5	0.082
LDL-C (mg/dL)	119.0 ± 27.0	123.0 ± 21.4	125.1 ± 26.1	0.248
Uric acid (mg/dL)	5.87 ± 1.01	5.94 ± 0.85	6.01 ± 1.17	0.665
Leukocyte count (cells/μL)	5556^a,b^ ± 1123	6210^a^ ± 1366	6530^b^ ± 1216	<0.001
hs-CRP (mg/L)	0.65^a^ ± 0.43	0.75 ± 0.43	0.86^a^ ± 0.72	0.029

*BMI* body mass index, *WC* waist circumference, *SBP* systolic blood pressure, *DBP* diastolic blood pressure, *FBS* fasting blood sugar, *HbA1c* glycated hemoglobin, *TG* triglycerides, *HDL-C* high-density lipoprotein cholesterol, *LDL-C* low-density lipoprotein cholesterol, *hs-CRP* high-sensitivity C-reactive protein

^*^values are mean ± standard deviation

^†^compared using by one-way analysis of variance

^a,b^same letters indicate statistical significance based on Bonferroni’s multiple comparison

### Correlation of inflammatory markers with clinical or biochemical parameters

In age-adjusted correlation analysis conducted on inflammatory markers and CVD risk factors, hs-CRP showed sforignificant positive correlations with BMI (*r* = 0.250, *p* < 0.001) and waist circumference (*r* = 0.248, *p* < 0.001) while leukocyte count showed significant positive correlations with BMI (*r* = 0.270, *p* < 0.001), waist circumference (*r* = 0.261, *p* < 0.001), SBP (*r* = 0.129, *p* = 0.045), DBP (*r* = 0.203, *p* = 0.002), HbA1c (*r* = 0.249, *p* < 0.001), TG (*r* = 0.142, *p* = 0.027), LDL-C (*r* = 0.190, *p* = 0.003), and uric acid (*r* = 0.233, *p* < 0.001). HDL-C showed significant negative correlations with both hs-CRP (*r* = −0.229, *p* < 0.001) and leukocyte count (*r* = −0.176, *p* = 0.006) (Table 3).

**Table 3:** Correlation of inflammatory markers with clinical or biochemical parameters

Variables	hs-CRP (mg/L)		Leukocyte count (cells/μL)	
	R	*p*-value^a^	R	*p*-value^a^
BMI (kg/m^2^)	0.250	<0.001	0.270	<0.001
WC (cm)	0.248	<0.001	0.261	<0.001
SBP (mmHg)	0.008	0.901	0.129	0.045
DBP (mmHg)	0.072	0.262	0.203	0.002
FBS (mg/dL)	0.040	0.531	0.089	0.167
HbA1c (%)	0.093	0.147	0.249	<0.001
TG (mg/dL)	0.069	0.285	0.142	0.027
HDL-C (mg/dL)	−0.229	<0.001	−0.176	0.006
LDL-C (mg/dL)	0.055	0.390	0.190	0.003
Uric acid (mg/dL)	0.121	0.060	0.233	<0.001

*BMI* body mass index, *WC* waist circumference, *SBP* systolic blood pressure, *DBP* diastolic blood pressure, *FBS* fasting blood sugar, *HbA1c* glycated hemoglobin, *TG* triglycerides, *HDL-C* high-density lipoprotein cholesterol, *LDL-C* low-density lipoprotein cholesterol, *hs-CRP* high-sensitivity C-reactive protein

^a^compared using age-adjusted partial correlation, R: correlation coefficient

### Work schedule and levels of inflammatory markers

After adjusting for variables potentially affecting inflammatory markers, hs-CRP was 0.92 ± 0.07 mg/L for current shift workers and 0.59 ± 0.06 mg/L for daytime workers (*p* = 0.002) while leukocyte count was 6,498 ± 144 cells/μL for current shift workers and 5,557 ± 124 cells/μL for daytime workers (*p* < 0.001); in both hs-CRP and leukocyte count, the average values (mean ± standard error) were significantly higher in current shift workers than in daytime workers. Leukocyte count was 6,264 ± 173 cells/μL for former shift workers and 5,557 ± 124 cells/μL for daytime workers and was significantly higher in former shift workers than in daytime workers (*p* = 0.004) (Table 4).

**Table 4:** Work schedule and levels of inflammatory markers

Work schedule group	hs-CRP (mg/L)		Leukocyte count (cells/μL)	
	Adjusted M ± SD^†^	*p*-value^*^	Adjusted M ± SD ^†^	*p*-value^*^
Daytime	0.59^a^ ± 0.06		5557^a,b^ ± 124	
Former shift	0.80 ± 0.08	0.002	6264^a^ ± 173	<0.001
Current shift	0.92^a^ ± 0.07		6498^b^ ± 144	

^†^compared using analysis of covariance, *M* mean, *SD* standard deviation

^*^adjusted for age, BMI, waist circumference, alcohol consumption, smoking, regular exercise, sleep duration, sleep debt, sleep insufficiency, education, and weekly work hours

^a,b^same letters indicate statistical significance based on Bonferroni’s multiple comparison

## Discussion

This cross-sectional study investigated the association between shift work and the inflammatory markers hs-CRP and leukocyte count, which are CVD predictors. Even after adjusting for variables that may affect hs-CRP level and leukocyte count, the values were still significantly higher in shift workers than in daytime workers.

The mechanism by which shift work increases CVD risk can potentially be attributed to the complex interaction between “psychosocial stress” due to job stress, decreased recovery from work, and work-life imbalance; “behavioral stress” due to sleep duration, sleep quality, smoking, nutrition, physical inactivity, and weight gain; and “physiological stress” exemplified by inflammation, blood coagulation, increased blood pressure due to the activation of the sympathetic nervous system, and hyper-activation of the hypothalamo-pituitary-adrenal axis. The interaction between these stresses could influence the development of atherosclerosis, metabolic syndrome, and type 2 diabetes, thereby potentially increasing the risk of CVD [30].

Inflammation is a common risk factor for atherosclerosis, metabolic syndrome, type 2 diabetes, and CVD [31]. CRP is an inflammatory marker and a typical acute phase reactant that increases with inflammation, infection, and trauma; it is produced in the liver in response to cytokines, such as interleukin-6 [19]. CRP induces complement activation, increases the uptake and oxidation of LDL-C, decreases nitric oxide production, induces tissue factor production, upregulates adhesion molecule expression, inhibits fibrinolysis through increased plasminogen activator inhibitor-1 expression, and promotes monocyte infiltration into the vascular wall, thereby playing an important role in atherosclerosis and CHD; furthermore, increased CRP is associated with metabolic syndrome, type 2 diabetes, and insulin resistance, and it is suggested to be an independent predictor of CVD in healthy individuals [19, 31]. Peripheral leukocyte count is also an indicator of inflammatory responses, and it increases in response to inflammatory cytokines produced during chronic inflammation. The increased leukocytes then activate the immune system and further increase the leukocyte count, leading to further cytokine production. Notably, increased leukocyte count is significantly correlated with the development of metabolic syndrome and CVD [32].

According to Sookoian et al., leukocyte count was significantly higher in shift workers than in daytime workers [33]; Puttonen et al. also reported an increase in hs-CRP in 3-shift workers and an increase in leukocyte count in 2-shift and 3-shift workers, thus agreeing with our findings [21]. Lu et al. recently showed that increased total and differential leukocyte counts (neutrophil, monocyte, and lymphocyte) were associated with shift work [34]; in contrast, Nam et al. reported that the total lymphocyte count decreased significantly in shift workers compared to that in daytime workers [35]. As the present study did not analyze differential leukocyte count, further investigation will be required in the future. Han et al. showed that shift work is associated with periodontitis, which may be an early indicator of severe inflammatory systemic disorder, supporting the results of the current study [36].

As mentioned above, inflammation plays an important role in the initiation and progression of atherosclerosis. Recent studies, which have used imaging techniques to show increased carotid intima media thickness in shift workers compared to daytime workers and have evaluated coronary artery calcium scoring to show that coronary artery disease risk increases in shift workers, have provided direct evidence for the association between shift work and atherosclerosis [37, 38].

Although not shown in the Results section, the duration of shift work and inflammatory markers did not have any significant correlation; the analysis was limited in that the shift worker sample was small and that the duration of shift work was concentrated to a certain period.

Shift workers often experience sleep disorders as they experience frequent disturbances in their waking and sleeping hours, thus disturbing the circadian rhythm [13]. Shorter or longer sleep duration and decreased sleep quality are associated with increased inflammatory markers and increased development of CVD [39]. A meta-analysis by Pilcher et al. showed that sleep duration shortens when shift workers have morning or night shifts, while it lengthens with evening shifts, and many studies have shown that night shift work was associated with reduced sleep duration and deteriorated sleep quality [40, 41]. In the present study, shift workers had longer sleep duration than daytime workers, although it did not reach statistical significance, while sleep debt and sleep insufficiency were significantly more severe. This may be attributed to the tendency of shift workers to supplement sleep debt caused by decreased sleep quality due to shift work [42]. However, this study is limited in terms of specific evaluation of the sleep quality, which should be considered in the future.

In terms of health-related behaviors, current shift workers did not exhibit significant differences between groups in alcohol consumption or regular exercise habits, while the smoking rate, a CVD risk factor, was significantly higher in current shift workers. Many studies have shown a higher smoking rate in shift workers than in daytime workers, and initiation of smoking was also related to shift work [30]. As smoking acts as a confounder and a mediator, including it as a covariate may dilute the actual effect of shift work on CVD [21]; however, it did not significantly influence the present results. However, the current study did not consider past smoking experience. This should be considered in future studies, as previous studies have shown contradictory results, showing both that leukocyte count was higher in ex-smokers than that in non-smokers even after cessation of smoking and that leukocyte count in ex-smokers decreased to a level similar to that of non-smokers after cessation of smoking [43, 44].

Aside from inflammatory markers, shift workers also showed higher levels of other CVD risk factors, including BP, FBS, and HbA1c, than those in daytime workers, corresponding with previous study results [11, 45]. Former shift workers also showed a significantly higher leukocyte count than daytime workers. Although Puttonen et al. showed that the prevalence of metabolic syndrome was higher in former shift workers than that in daytime workers, another study showed no differences in inflammatory markers between groups; this was explained by the observation that inflammatory markers increased during shift work but tended to normalize after leaving shift work [21, 46]. However, Guo et al. showed that retired individuals with past shift work experience of 10 years or more had higher risks of hypertension and diabetes than those without history of shift work; accordingly, it is difficult to estimate the continuous influence of shift work after its cessation [47]. As mentioned earlier, considering the reversible changes in the inflammatory markers after quitting shift work, there might be some differences in the inflammatory markers between former shift workers who have performed shift work in recent years and former shift workers who have not performed shift work in recent years, even if their duration of shift work was the same. In a study by Titova et al., former shift work was categorized according to a criterion of 5 years: past shift workers were those who did not perform shift work during the past 5 years but had worked night shifts more than 5 years ago, and recent former shift workers were defined as those who performed shift work during the past 5 years but not at the time of data collection; greater impairment of cognitive function was observed in current and recent former shift workers than in non-shift workers, while there was no significant difference between non-shift workers and past shift workers [48]. Because most studies on the association between shift work and CVD did not present clear-cut criteria for former shift worker, future studies need to take this into consideration. Moreover, former shift workers who performed shift work for a long duration might show similar levels of inflammatory markers as current shift workers until reversible changes appear. In the present study, there was no statistically significant difference in inflammatory markers between current and former shift workers. In a recent study by Vetter et al., a dose-response relationship between the duration of shift work and the risk of CHD was observed; moreover, after shift work ceased, the risk of CHD decreased over time [49]. However, it is still unknown at what point being exposed to shift work is still safe with respect to the risk of CVD and how much time is required for the reversal process to show recovery back to the original state. As the present study lacked a detailed investigation of former shift workers, future studies should consider shift work characteristics of former shift workers and the length of time that has passed since cessation.

There are several limitations of the present study. First, this was a cross-sectional study and thus it was difficult to investigate the causal relationship between shift work and inflammatory markers. Second, the healthy worker effect might have been present where the characteristics of shift work leads healthier workers to be employed in shift work rather than daytime work, causing an underestimation of the effect of shift work on health. Third, there was no consideration of the previously mentioned various confounders that may contribute to shift work-related development of CVD, such as psychosocial stress, including income and job stress; behavioral stress, including nutrition and sleep quality; and occupational characteristics, including tenure, work intensity, and hazardous materials handled at work. Fourth, although this study focused on manual workers, a more homogenous population should be selected for future studies as each of our participants had different work content, environment, and exposure to hazardous materials, as well as different work schedules according to their education level. Fifth, this study did not establish a detailed definition of former shift workers; thus, additional studies may be required to confirm the differences in inflammatory markers between past shift workers and recent former shift workers and the reversibility of shift work-induced adverse health effects.

Despite the limitations, this study holds significance in that it was the first South Korean study revealing an association between shift work and inflammatory markers; moreover, this study attempted to understand the effects of shift work on health through easily testable indicators. The hs-CRP level and leukocyte count were higher in shift workers within the normal ranges; nonetheless, the results of this study confirmed positive correlations between inflammatory markers and CVD risk factors. Further, previous studies reported increased risks of CVD with increased levels of inflammatory markers, an increase within the normal range can be interpreted as a significant result per se [20, 32, 50, 51].

As the current participants were relatively young (<40 years of age) and considering that CVD is a progressive disease with a long subclinical phase, regular follow-up of inflammatory markers should be conducted to allow early detection of the effects of shift work on health.

## Conclusions

In conclusion, this study confirmed an association between shift work and increases in inflammatory markers. Considering that multiple previous studies have reported an association between increased inflammatory markers and CVD, follow-up concerning inflammatory markers would be helpful for the prevention of CVD in shift workers. A well-designed prospective study including a large sample size that compensates for the aforementioned limitations could help determine the causal relationship between shift work and inflammatory markers.

## Abbreviations

BMI: Body mass index
CHD: Coronary heart disease
CVD: Cardiovascular disease
DBP: Diastolic blood pressure
FBS: Fasting blood sugar
HbA1c: Glycated hemoglobin
HDL-C: High-density lipoprotein cholesterol
hs-CRP: High-sensitivity C-reactive protein
LDL-C: Low-density lipoprotein cholesterol
MI: Myocardial infarction
SBP: Systolic blood pressure
TG: Triglycerides

## References

[1] Lee KJ, Kim JJ (2008). Relationship of shift work to cardiovascular and gastrointestinal symptoms in Korean female workers. Korean J Occup Environ Med.

[2] Park TJ, Paek DM, Joh KO, Park JS, Cho SI (2012). The relationship between shift work and work-related injuries among Korean workers. Korean J Occup Env Med.

[3] Szosland D (2010). Shift work and metabolic syndrome, diabetes mellitus and ischaemic heart disease. Int J Occup Med Environ Health.

[4] Ker K, Edwards PJ, Felix LM, Blackhall K, Roberts I (2010). Caffeine for the prevention of injuries and errors in shift workers. Cochrane Database Syst Rev.

[5] IARC (2010). IARC monographs on the evaluation of carcinogenic risks to humans. VOLUME 98 painting, firefighting, and shiftwork.

[6] European Foundation for the Improvement of Living and Working Conditions (2015). First findings: 6th European working conditions survey.

[7] Bae KS, Park TJ, Lee MH, Lee YH, Kim JJ, Jung SK (2013). Shift system and working hours.

[8] Bøggild H, Knutsson A (1999). Shift work, risk factors and cardiovascular disease. Scand J Work Environ Health.

[9] Vyas MV, Garg AX, Iansavichus AV, Costella J, Donner A, Laugsand LE (2012). Shift work and vascular events: systematic review and meta-analysis. BMJ.

[10] Brown DL, Feskanich D, Sanchez BN, Rexrode KM, Schernhammer ES, Lisabeth LD (2009). Rotating night shift work and the risk of ischemic stroke. Am J Epidemiol.

[11] De Bacquer D, Van Risseghem M, Clays E, Kittel F, De Backer G, Braeckman L (2009). Rotating shift work and the metabolic syndrome: a prospective study. Int J Epidemiol.

[12] Bara AC, Arber S (2009). Working shifts and mental health – findings from the British household panel survey (1995–2005). Scand J Work Environ Health.

[13] Akerstedt T (2003). Shift work and disturbed sleep/wakefulness. Occup Med.

[14] Knutsson A, Bøggild H (2010). Gastrointestinal disorders among shift workers. Scand J Work Environ Health.

[15] Knutsson A, Alfredsson L, Karlsson B, Akerstedt T, Fransson EI, Westerholm P (2013). Breast cancer among shift workers: results of the WOLF longitudinal cohort study. Scand J Work Environ Health.

[16] Kubo T, Ozasa K, Mikami K, Wakai K, Fujino Y, Watanabe Y (2006). Prospective cohort study of the risk of prostate cancer among rotating-shift workers: findings from the Japan collaborative cohort study. Am J Epidemiol.

[17] Knutsson A, Akerstedt T, Jonsson BG, Orth-Gomer K (1986). Increased risk of ischaemic heart disease in shift workers. Lancet.

[18] Ross R (1999). Atherosclerosis – an inflammatory disease. N Engl J Med.

[19] Shrivastava AK, Singh HV, Raizada A, Singh SK (2015). C-reactive protein, inflammation and coronary heart disease. Egypt Heart J.

[20] Madjid M, Awan I, Willerson JT, Casscells SW (2004). Leukocyte count and coronary heart disease implications for risk assessment. J Am Coll Cardiol.

[21] Puttonen S, Viitasalo K, Harma M (2011). Effect of shiftwork on systemic markers of inflammation. Chronobiol Int.

[22] Franceschi C, Campisi J (2014). Chronic inflammation (inflammaging) and its potential contribution to age-associated diseases. J Gerontol A Biol Sci Med Sci.

[23] Strohacker K, Wing RR, McCaffery JM (2013). Contributions of body mass index and exercise habits on inflammatory markers: a cohort study of middle-aged adults living in the USA. BMJ Open.

[24] Volpato S, Pahor M, Ferrucci L, Simonsick EM, Guralnik JM, Kritchevsky SB (2004). Relationship of alcohol intake with inflammatory markers and plasminogen activator inhibitor-1 in well-functioning older adults: the health, aging, and body composition study. Circulation.

[25] Lee J, Taneja V, Vassallo R (2012). Cigarette smoking and inflammation: cellular and molecular mechanisms. J Dent Res.

[26] Altman NG, Izci-Balserak B, Schopfer E, Jackson N, Rattanaumpawan P, Gehrman PR (2012). Sleep duration versus sleep insufficiency as predictors of cardiometabolic health outcomes. Sleep Med.

[27] Prather AA, Marsland AL, Hall M, Neumann SA, Muldoon MF, Manuck SB (2009). Normative variation in self-reported sleep quality and sleep debt is associated with stimulated pro-inflammatory cytokine production. Biol Psychol.

[28] Friedman EM, Herd P (2010). Income, education, and inflammation: differential associations in a national probability sample (The MIDUS study). Psychosom Med.

[29] Nakanishi N, Suzuki K, Tatara K (2003). Association between lifestyle and white blood cell count: a study of Japanese male office workers. Occup Med.

[30] Puttonen S, Harma M, Hublin C (2010). Shift work and cardiovascular disease–pathways from circadian stress to morbidity. Scand J Work Environ Health.

[31] Ridker PM, Buring JE, Cook NR, Rifai N (2003). C-reactive protein, the metabolic syndrome, and the risk of incident cardiovascular events: an 8-year follow-up of 14719 initially healthy American women. Circulation.

[32] Lee SH, Kim SM (2013). The relationship between the increase of white blood cell and development of hyperglycemia among healthy adult males. Korean J Fam Pract.

[33] Sookoian S, Gemma C, Fernandez Gianotti T, Burgueno A, Alvarez A, Gonzalez CD (2007). Effects of rotating shift work on biomarkers of metabolic syndrome and inflammation. J Intern Med.

[34] Lu LF, Wang CP, Tsai IT, Hung WC, Yu TH, Wu CC (2016). Relationship between shift work and peripheral total and differential leukocyte counts in Chinese steel workers. J Occup Health.

[35] Nam M, Joe SH, Jung IK, Soh KY, Chung CK (1997). Anxiety, depression and immune functions of shift workers. Korean J Occup Med.

[36] Han DH, Khang YH, Jung Choi K, Lim S (2013). Association between shift work and periodontal health in a representative sample of an Asian population. Scand J Work Environ Health.

[37] Puttonen S, Kivimaki M, Elovainio M, Pulkki-Raback L, Hintsanen M, Vahtera J (2009). Shift work in young adults and carotid artery intima-media thickness: the cardiovascular risk in young Finns study. Atherosclerosis.

[38] Kang W, Park WJ, Jang KH, Kim SH, Gwon DH, Lim HM (2016). Coronary artery atherosclerosis associated with shift work in chemical plant workers by using coronary CT angiography. Occup Environ Med..

[39] Dowd JB, Goldman N, Weinstein M (2011). Sleep duration, sleep quality, and biomarkers of inflammation in a Taiwanese population. Ann Epidemiol.

[40] Pilcher JJ, Lambert BJ, Huffcutt AI (2000). Differential effects of permanent and rotating shifts on self-report sleep length: a meta-analytic review. Sleep.

[41] Garde AH, Hansen AM, Hansen J (2009). Sleep length and quality, sleepiness and urinary melatonin among healthy Danish nurses with shift work during work and leisure time. Int Arch Occup Environ Health.

[42] Lee JT, Lee KJ, Park JB, Lee KW, Jang KY (2007). The relations between shiftwork and sleep disturbance in a University hospital nurses. Korean J Occup Environ Med.

[43] Petitti DB, Kipp H (1986). The leukocyte count: associations with intensity of smoking and persistence of effect after quitting. Am J Epidemiol.

[44] Sunyer J, Munoz A, Peng Y, Margolick J, Chmiel JS, Oishi J (1996). Longitudinal relation between smoking and white blood cells. Am J Epidemiol.

[45] Suwazono Y, Dochi M, Oishi M, Tanaka K, Kobayashi E, Sakata K (2009). Shiftwork and impaired glucose metabolism: a 14-year cohort study on 7104 male workers. Chronobiol Int.

[46] Puttonen S, Viitasalo K, Harma M (2012). The relationship between current and former shift work and the metabolic syndrome. Scand J Work Environ Health.

[47] Guo Y, Liu Y, Huang X, Rong Y, He M, Wang Y (2013). The effects of shift work on sleeping quality, hypertension and diabetes in retired workers. PLoS One.

[48] Titova OE, Lindberg E, Elmståhl S, Lind L, Schiöth HB, Benedict C (2016). Association between shift work history and performance on the trail making test in middle-aged and elderly humans: the EpiHealth study. Neurobiol Aging.

[49] Vetter C, Devore EE, Wegrzyn LR, Massa J, Speizer FE, Kawachi I (2016). Association between rotating night shift work and risk of coronary heart disease among women. JAMA.

[50] Ridker PM (2001). High-sensitivity C-reactive protein: potential adjunct for global risk assessment in the primary prevention of cardiovascular disease. Circulation.

[51] Ridker PM (2003). Clinical application of C-reactive protein for cardiovascular disease detection and prevention. Circulation.

